# Gene discovery in the horned beetle *Onthophagus taurus*

**DOI:** 10.1186/1471-2164-11-703

**Published:** 2010-12-14

**Authors:** Jeong-Hyeon Choi, Teiya Kijimoto, Emilie Snell-Rood, Hongseok Tae, Youngik Yang, Armin P Moczek, Justen Andrews

**Affiliations:** 1 Center for Genomics and Bioinformatics, Indiana University, Bloomington, Indiana, 47405, USA; 2 Department of Biology, Indiana University, Bloomington, Indiana, 47405, USA; 3 School of Informatics and Computing, Indiana University, Bloomington, Indiana 47408, USA

## Abstract

**Background:**

Horned beetles, in particular in the genus *Onthophagus*, are important models for studies on sexual selection, biological radiations, the origin of novel traits, developmental plasticity, biocontrol, conservation, and forensic biology. Despite their growing prominence as models for studying both basic and applied questions in biology, little genomic or transcriptomic data are available for this genus. We used massively parallel pyrosequencing (Roche 454-FLX platform) to produce a comprehensive EST dataset for the horned beetle *Onthophagus taurus*. To maximize sequence diversity, we pooled RNA extracted from a normalized library encompassing diverse developmental stages and both sexes.

**Results:**

We used 454 pyrosequencing to sequence ESTs from all post-embryonic stages of *O. taurus. *Approximately 1.36 million reads assembled into 50,080 non-redundant sequences encompassing a total of 26.5 Mbp. The non-redundant sequences match over half of the genes in *Tribolium castaneum*, the most closely related species with a sequenced genome. Analyses of Gene Ontology annotations and biochemical pathways indicate that the *O. taurus *sequences reflect a wide and representative sampling of biological functions and biochemical processes. An analysis of sequence polymorphisms revealed that SNP frequency was negatively related to overall expression level and the number of tissue types in which a given gene is expressed. The most variable genes were enriched for a limited number of GO annotations whereas the least variable genes were enriched for a wide range of GO terms directly related to fitness.

**Conclusions:**

This study provides the first large-scale EST database for horned beetles, a much-needed resource for advancing the study of these organisms. Furthermore, we identified instances of gene duplications and alternative splicing, useful for future study of gene regulation, and a large number of SNP markers that could be used in population-genetic studies of *O. taurus *and possibly other horned beetles.

## Background

Horned beetles, in particular in the genus *Onthophagus*, are important models for studies on sexual selection [1-3], biological radiations [4-7], endocrine regulation of development [8-11], biological control of invasive species [12-14], conservation biology [15,16], and forensic biology [17-19]. *Onthophagus *beetles have more recently gained particular prominence as models for studying the origin and diversification of novel traits (hundreds of species express diverse horns and horn-like structures that lack obvious homology to any other traits in insects [20,21]) and the developmental underpinnings of phenotypic plasticity (species adjust adult morphology, behavior, and physiology in response to larval nutrition, ranging from subtle adjustment to profound modifications depending on species and phenotype [22-27]).

Despite their growing prominence as models for studying both basic and applied questions in biology, no genome projects exist for any *Onthophagus *species. Instead, investigations into the genetic basis of *Onthophagus *biology have had to rely on homology-based gene-by-gene cloning [28,29] and only very recently on low throughput EST sequencing [30]. At the same time, development of genomic resources in several other insect models, such as *Drosophila*, mosquitoes, *Tribolium *beetles [31], honey bees [32], and several lepidopteran species [33-35], has greatly advanced insights into the molecular and developmental genetics, physiology, and evolution of these organisms. *Onthophagus *beetles offer great opportunities to add to the study of important biological phenomena pioneered through the study of these earlier models, such as the regulation of arthropod development, which has been informed in large part through work on fruit flies and *Tribolium *beetles [36,37], the origin of novel complex traits, as studied in butterfly wing patterns [38,39], or the genetic regulation of nutrition-sensitive development, a central focus of honey bee research [40-42].

Furthermore, several other experimental techniques and tools have been successfully developed for *Onthophagus*, most notably RNAinterference mediated transcript depletion [43]. Applying such tools to the study of *Onthophagus *biology has, however, been hampered by the paucity of candidate genes and pathways available for investigation. The very recent development of modest EST resources for *Onthophagus taurus *using traditional Sanger sequencing [30] has already facilitated several important new research efforts [44-46]. Combined, this suggests that studies on *Onthophagus *beetles are poised to make rapid progress once large-scale genomic or transcriptomic resources are available, which in turn promises to advance our understanding of fundamental and applied question in evolution and developmental biology. Here we describe an EST collection developed for the horned beetle *Onthophagus taurus*, the most commonly studied species of horned beetle to date.

## Results

### Sequencing, assembly and analysis of non-redundant sequences

We wished to sample a broad diversity of transcribed sequences including those expressed during the elaboration of horns. Given that in *Drosophila melanogaster*, which shares holometabolous development with beetles, the greatest number of genes are expressed in adult females, followed by pupal and then larval stages [47], we anticipated that sampling transcription from post-embryonic stages of *Onthophagus *would allow us to obtain a broad diversity of expressed sequences. We prepared sequencing libraries using RNA isolated from all post-embryonic life stages including both sexes (see Methods). Sequencing using the 454 GS FLX titanium platform yielded 1,366,749 sequence reads. After cleaning, 1,361,424 reads (average length: 440 nt) were assembled using Newbler [48] and MIRA [49], resulting in 39,088 contigs (average length: 583 nt, average coverage: 24 reads) and 10,992 singletons (average length: 337 nt) (Table 1). Thus, the approximately 1.36 million reads collapsed into 50,080 "non-redundant" sequences totaling 26,520,165 nt. The sequence reads are available at NCBI Sequence Read Archive (SRA010107) and the assembled sequences are provided in Additional file 1.

**Table 1:** Sequencing and assembly statistics

Category	
Total number of reads	1,366,749
Total length of reads (bp)	625,825,203
Total number of reads cleaned	1,361,424
Total length of reads cleaned (bp)	598,655,879
Number of reads placed	1,302,023
Number of singletons	10,992
Total length of singletons (bp)	3,714,066
Average length of singletons (bp)	337
Largest singleton (bp)	692
Number of contigs	39,088
Total length of contigs (bp)	22,806,009
Average length of contigs (bp)	583
Largest contig (bp)	6,401
Average read coverage of contigs	24

**Figure 1:**
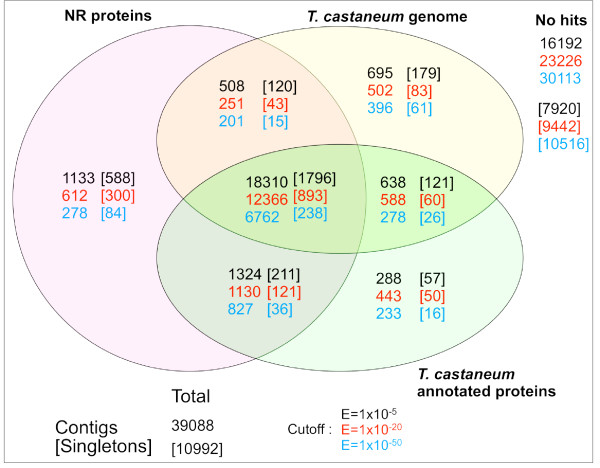
**Sequence matches to NR protein database and *Tribolium *genome and proteins**. Venn diagram showing the number of *Onthophagus *contigs and singletons (in parenthesis) with sequence matches against the NCBI NR database [52], *Tribolium *genome sequence [92] and *Tribolium *annotated proteins [92]. The number of sequence matches at E-value cut-offs of 1 × 10^-5^, 1 × 10 ^-20 ^and 1 × 10^-50 ^are shown in black, red and blue, respectively.

We characterized the non-redundant sequences in terms of similarity to known repeated sequences, known protein-coding sequences, and known transcribed sequences. First, running RepeatMasker [50] with RepBase database for *Drosophila *[51] identified 37 LINEs, 39 LTR elements, 41 DNA transposons and 67 small RNAs (Additional file 2). In total, 1.05% of base pairs were masked including 33 kbp of simple repeats and 206 kbp of low complexity regions. Second, aligning the *Onthophagus *non-redundant sequences with the NCBI non-redundant (NR) protein sequence database [52], revealed that 54% (21,275) of the contigs had sequence matches with known proteins with an E-value < 1 × 10^-5^, 37% (14,359) had matches with an E-value < 1 × 10^-20^, and 21% (8,068) had matches with an E-value < 1 × 10^-50 ^(Table 2). Given that, on average, singletons were shorter than contigs, both in terms of overall length and the length of predicted open reading frames (Table 1), it was to be expected that they would be less likely to include coding sequence. Indeed, 25% (2,715), 12% (1,357) and 3% (373) of singletons had matches with E-values of < 1 × 10^-5^, <1 × 10^-20^, and <1 × 10^-50^, respectively. While the singletons had proportionately fewer protein matches, they do contribute significantly to the information content of the non-redundant sequences. For instance, approximately 14% of the NR protein sequences with matches (E-value < 1 × 10^-5^) against the non-redundant sequences matched exclusively with singletons. Third, we aligned the non-redundant sequences against databases of sequences from *T. castaneum*, the only other beetle for which comprehensive sequence data is available. This revealed that a small proportion of the non-redundant sequences that do not have matches against NR do have matches against the *T. castaneum *genome or annotated proteins (Figure 1). Approximately 4% (1,621) of the contig sequences, and 3% (357) of the singletons are in this category (E-value < 1 × 10^-5^).

**Table 2:** Sequence matches against public databases

Database	E-value	Contigs		Singletons		Total	
		
		Query	Subject	Query	Subject	Query	Subject
NCBI NR	10^-5^	21,275	12,739	2,715	2,371	23,990	14,223
	10^-20^	14,359	9,604	1,357	1,261	15,716	10,394
	10^-50^	8,068	6,350	373	356	8,441	6,574

*Tribolium *unigene	10^-5^	14,807	5,158	3,448	1,760	18,255	5,322
	10^-20^	10,260	4,671	2,236	1,259	12,946	4,799
	10^-50^	5,945	3,791	1,011	624	6,956	3,880

*Tribolium *annotated proteins	10^-5^	20,560	8,911	2,185	1,614	22,745	9,303
	10^-20^	14,497	7,888	1,124	951	15,621	8,203
	10^-50^	8,100	5,798	316	284	8,416	5,907

The total numbers of *Onthophagus *sequences with matches against public databases at the indicated E-value cut-off. Databases: NCBI NR [52], *Tribolium *UniGene [91], and *Tribolium *proteins [92]. "Query" denotes the total number of *Onthophagus *sequences with matches against sequences from the database at the indicated cut-off. "Subject" denotes the total number of sequences from the indicated database with matches against *Onthophagus *sequences at the indicated E-value cut-off.

Further analyses of these sequences revealed the following: (i) 759 sequences matched the *T. castaneum *annotated protein coding sequences and genome. (ii) 345 sequences matched the *T. castaneum *annotated proteins but not the *T. castaneum *genome, and thus are likely to be genuine transcribed sequences, but the homologous sequences may not be included in the assembled *T. castaneum *genome. (iii) Of 874 sequences that matched only the *T. castaneum *genome, 446 contigs and 114 singletons matched *T. castaneum *sequences that lie within the bounds of annotated genes, but are not annotated as transcribed sequences. These may represent sequences that are included in mature transcripts in *O. taurus *but not *T. castaneum*. Alternatively, they may identify sequences that are included in mature transcripts in *T. castaneum *but not annotated as such. (iv) 314 sequences matched unannotated genomic sequence in *T. castaneum*, and thus may identify lineage specific genes, or more likely, genes that are not annotated in *T. castaneum*. In total then, 52% (25,968) of the *O. taurus *non-redundant sequences matched either NR, or *T. castaneum *genomic or protein sequences. Conversely, the *O. taurus *non-redundant sequences match with approximately 56% (9,303/16,645) of the gene models in *T. castaneum, *and 59% (5,322/9,053) of the sequence clusters in the *Tribolium *UniGene set (Table 2). These data suggests we have sampled a significant fraction of the *O. taurus *transcriptome.

**Figure 2:**
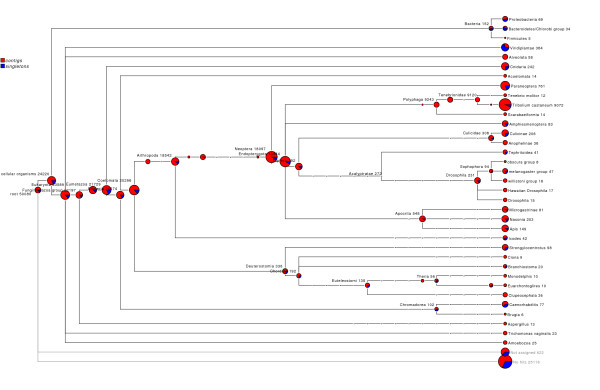
**Taxonomic distribution of sequence matches**. Phylogenetic tree showing the number of *O. taurus *non-redundant sequences assigned to branches. The MEGAN algorithm used in this analysis assigns each sequence to the lowest common ancestor of the set of taxa with corresponding sequence matches. The total numbers of *O. taurus *sequences assigned to each branch are indicated in decimals and by the pie chart area (Log scale). Pie graph colors indicate the proportion of contigs (red) and singeltons (blue) assigned to each branch.

Although we took measures to minimize gut contamination (see Methods), the animals used to construct the library had to be cultured in cow dung, which is rich in plant and microbial material. We consequently anticipated that our EST might include some non-*Onthophagus *sequences, and therefore explored the taxonomic distribution of sequences matching the *Onthophagus *non-redundant sequences. We did this using MEGAN [53], which assigns each sequence to the lowest common ancestor of the set of taxa with corresponding sequence matches. This analysis revealed that, of the sequences that had sequence matches and were assigned, the majority were assigned to the expected taxonomic groups within the Arthropoda (Figure 2). Specifically, 8,462 contigs and 611 singletons, were classified to *Tribolium*: 9,120 to Tenebrionidae, 9,243 to Polyphaga, 13,944 to Endopterygota, 18,097 to Neoptera, 18,542 to Arthropoda, 20,296 to Coelomata, 20,674 to Bilateria, 21,981 to Metazoa, and 23,589 to Eukaryota. Reassuringly, a relatively small proportion was assigned to taxa outside beetles. For instance only 83 contigs and 69 singletons were classified to bacteria (Figure 2). This indicates that *O. taurus *sequences are not significantly contaminated by ESTs from bacteria or plants.

Nevertheless, 48% of the *O. taurus *non-redundant sequences did not match with the *T. castaneum *genome or proteins. Of 38,050 contigs, 21,847 sequences had significant hits (E-value cutoff of 1 × 10^-5^) to the *Tribolium *genome, *Tribolium *proteins, and/or NCBI NR (Figure 1), while 16,203 sequences had no significant match to any of these databases. We performed additional analyses on the "no-hit" sequences to determine whether they represent poor sampling (i.e., short or few reads) or biologically interesting (highly divergent) genes. Compared to sequences with significant hits, the no-hit sequences had shorter contig lengths (mean (SE): hit = 733.5 (3.17); no hit = 391.1 (3.69); *F*_*38048 *_= 4941, *P *= 0), smaller proportions of read length made up of predicted coding sequence (mean (SE): hit = 0.751 (0.002); no hit = 0.518 (0.002); *F*_*38048 *_= 7,737, *P *= 0), and fewer reads (mean (SE): hit = 74.1 (0.67); no hit = 22.64 (0.78); *F*_*38048 *_= 2,492, *P *= 0). While the mean distribution of contig length, proportion of coding sequence and read number was significantly different between hit and no hit sequences, there was considerable overlap between the two distributions (see Additional file 3). In particular, 21% of the no hit sequences (3,338 of 16,203) had at least the average read length (733 bp) and proportion coding sequence (0.74) of sequences with hits. This subset of high quality sequences had on average 39 reads, suggesting they were not simply genes with low expression. Taken together, this analysis suggests that while many of our "no hit" sequences likely represent low information content of a contig due to short or few reads, a significant proportion of these no hit sequences may represent highly divergent or novel genes that may prove interesting in future study.

In summary, the *O. taurus *non-redundant sequences match with over half of the genes in *T. castaneum*. If we assume that these two beetle species have similar total gene numbers, then we can infer that we have sampled a significant proportion of genes in *O. taurus*. There is no evidence that the *O. taurus *sequences are significantly contaminated with sequences from other taxa. Furthermore, we seem to have sampled many genes that may represent highly divergent or novel proteins.

### Clustering related sequences

It was to be expected that many of the non-redundant sequences would derive from non-overlapping regions of common transcripts. Indeed the 23,990 non-redundant *Onthophagus *sequences match a total of 14,223 distinct sequences in the NCBI NR protein database (Table 2). We took two approaches to identify clusters of non-redundant sequences that potentially derive from common transcription units.

**Figure 3:**
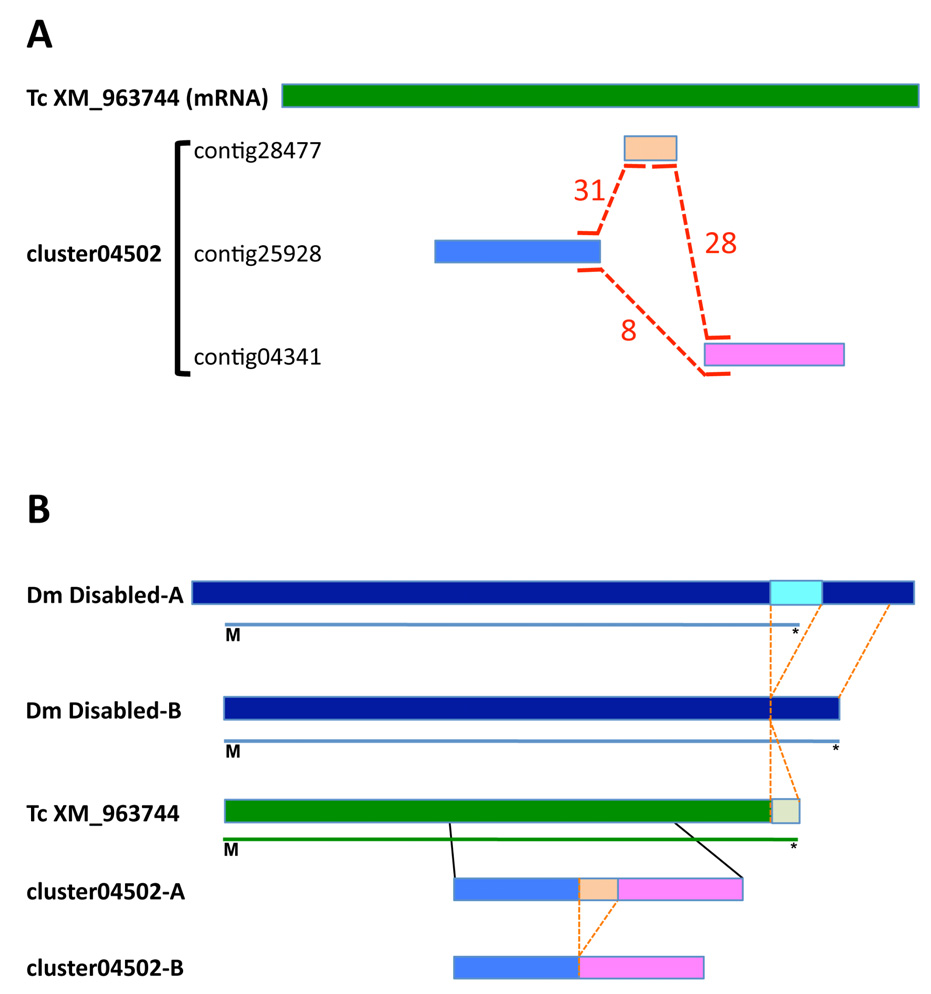
**Bi-connected components and alternative splicing**. **A**. An example of a bi-connected component structure (BCCs). A BBC composed of three contigs 28477, 25928 and 04341 that share three independent sets of 31, 28, and 8 broken reads, respectively (indicated by dashed line) in relation to the homologous *T. castaneum *transcript (Tc XM963744). Our analysis suggests that this pattern is reflective of two alternative splice variants present in the *Onthophagus *transcriptome. **B**. The three conceptual polypeptide sequences from these contigs align to a contiguous region of the *Disabled *protein from *Tribolium*, supporting this hypothesis. Shown are, from top to bottom, two alternative *Drosophila Disabled *transcripts (dark blue lines; thin light blue lines indicate first methionine (M) and stop codon (*)), the homologous *Tribolium *sequence (green; no alternative transcripts are known from *Tribolium*) and the relative positions of contigs 28477, 25928 and 04341. Note that the contig 28477 (light orange), which based on our analysis is a putatively alternatively spliced exon, does not share similarity with the exon that is alternatively spliced in *Drosophila*.

The first approach to clustering the non-redundant sequences was based on an analysis of "broken reads", or individual sequence reads that were placed in two or more contigs during the assembly of contigs. Pairs of contigs may be linked by broken reads if they (i) derive from the same gene but fail to assemble due to sequence polymorphisms between alleles, (ii) derive from alternatively spliced transcripts, (iii) derive from recently duplicated genes that still include some sequence similarity, or (iv) if the read(s) come from chimeric clones. In order to identify groups of contigs that are linked by broken reads we created a graph in which contigs are represented as nodes and broken reads represented as edges connecting nodes. This identified 5,136 *connected components *(CCs, subgraphs in which the nodes are connected by paths of edges), including 2,603 *bi-connected components *(BCCs, subgraphs that are not split of any one edge is removed, Additional file 4). The BCCs identify groups of three or more contigs that are linked by *independent *broken sequence reads. Figure 3A shows an example of a simple BCC composed of three contigs that share three independent sets of broken reads. The likelihood of BCCs resulting from chimeric clones is extremely low, as the minimum BCC of three nodes, would require three chimeras linking the three genes to occur independently. We reasoned that contigs with BCCs arising from different genomic origins would share different levels of sequence similarity - contigs from divergent alleles would have higher sequence similarity than contigs derived from duplicated genes, and contigs derived from alternatively spliced exons may share no sequence similarity at all. We therefore performed *inter se *Blastn sequence alignments of the contigs within each BCC, and categorized the BCCs as follows. First, pairs of contig sequences that had at least 50 bp of at least 95% sequence identity, flanked on either side by no more than 10 nt of less than 95% sequence identity (single stranded overhangs of >10 nt were permitted), were flagged as putative alleles and merged. Second, pairs of contigs that failed to meet the criteria for allelic variants and had Blastn matches of E-value < 1 × 10^-5 ^were flagged as putative duplicates. Third, pairs of contigs that failed to meet the criteria for allelic variants, and did not have Blastn matches of E-value < 1 × 10^-5 ^were flagged as putatively derived from alternative splicing. An analysis of known duplicated and alternatively spliced genes in *Drosophila *suggests that the use of this method cannot fully exclude misclassification, but that the frequency of false calls should overall be low (see Methods). Therefore while the BCCs provide strong evidence that contigs are related, the classification based on sequence similarity is only suggestive.

A total of 4,205 contigs met the first set of criteria and were flagged as putative alleles and merged into 2,026 groups. The hypothesis that these contigs derive from the same gene was supported by sequence matches against NR proteins. Of the pairs of merged contigs where both contigs had sequence matches against NR, 99% (1,547/1,565) had best matches to the same protein sequence, and only 1% (18/1,565) matched different proteins.

A total of 85 pairs of contigs met the second set of criteria and were flagged as presumptive duplicates. The hypothesis that these contigs derive from duplicate genes was supported by sequence matches against NR proteins. Of the pairs of contigs where both contigs have sequence matches against NR, 96% (23/24) had best matches to the same NR protein, and only 4% (1/24) had best matches to different proteins. We also asked whether there is evidence that the genes corresponding to the NR proteins are likely to be duplicated in the genomes of related species. Of the sequences tested 85% had two matches (E-value < 1 × 10^-5^) in *T. castaneum *(of 26 with at least one match) and 83% had two matches in a *D. melanogaster *(of 24 with at least one match). This indicates that 83-85% of the BCCs flagged as putatively duplicated in *Onthophagus *are also duplicated in other arthropods providing support for this classification.

We identified 753 groups of contigs (BCC) that met the third set of criteria and were thus flagged as putatively derived from alternative splicing. The hypothesis that these BCCs derive from alternatively spliced transcripts was supported by sequence matches against NR proteins, and *Drosophila *genes. Of these BCCs in which all contigs had sequence matches against NR, 62% (138/221) were composed of contigs that all had best matches with the same NR protein. We also investigated whether the corresponding genes (best Blastp match with a minimum of E-value = 1 × 10^-5^) in *D. melanogaster *are annotated as being alternatively spliced. 67% of the 206 cases tested were annotated as alternatively spliced in *D. melanogaster*. These data support the prediction that the corresponding genes are indeed alternatively spliced in *O. taurus*. A simple example is illustrated in Figure 3. Contigs 28477 (108nt), 25928 (769nt) and 04341 (639nt) form a BCC that is joined by a total of 67 broken reads (Figure 3A). Each edge of the BCC is defined by multiple broken reads, with the minimum being eight. The three contigs do not share significant sequence similarity among themselves (5.9% translated amino acid sequence identity between contig 25928 vs. contig 28477, 10.8% between contig 28477 vs. contig 04341, and 18.2% between contig 25928 vs. contig 04341), and as such, were flagged in our analysis as putatively belonging to a common transcription unit with alternative splicing. Indeed, the three conceptual polypeptide sequences from these contigs align to a contiguous region of the Disabled protein from *T. castaneum*, supporting this hypothesis. It should be noted that many of the BCCs that putatively result from alternative splicing have complex structures the resolution of which will require sequencing genomic and/or cDNA sequences. In summary, the analysis of BCCs grouped 10,387 of the non-redundant sequences into 2,603 groups flagged as putatively derived from a common gene (2,026 as allelic variants and 753 as alternatively spliced). While the BCC analysis provides reasonably strong evidence for merging sequences, it clearly does not capture all likely cases, and we therefore turned to similarity to known genes as a more comprehensive means of grouping non-redundant sequences, as described next.

In the second approach to clustering *Onthophagus *sequences we used similarity to sequences in the HomoloGene database [54], which is composed of groups of homologous gene sequences from 20 sequenced eukaryotic genomes. *Onthophagus *sequences that match the same HomoloGene sequence are likely to either derive from a single gene, or closely related gene family, in the *Onthophagus *genome. A total of 18,976 non-redundant *Onthophagus *sequences (17,160 contigs and 1,807 singletons) matched sequences from a total of 12,464 HomoloGene groups with an E-value < 1 × 10^-5 ^(Table 3, Additional file 5). At this stringency, 55% (6,839) of the clusters are composed of two or more *Onthophagus *sequences (14,062 contigs and 1,496 singletons total) and the remaining 45% (5,625) are composed of individual *Onthophagus *sequences (4,101 contigs and 512 singletons). Restricting the clustering only to cases where all of the *Onthophagus *sequences within a cluster have the best hit to the *same *HomoloGene sequence (shown in parenthesis in Table 3, and referred to here as "major clusters") reduced the total number of clusters from 12,464 clusters (E-value < 1 × 10^-5^) to 8,504 clusters (E-value < 1 × 10^-5^) (Table 3). There are 12,708 contigs and 1,160 singletons assigned uniquely to 6,839 HomoloGene groups. The number of clusters falls to 8,524 at E-value < 1 × 10^-20^, and then to 4,846 at E-value < 1 × 10^-50^. The major clusters represent the highest confidence set of non-redundant and presumably protein coding sequences. Thus, the 1.36 million reads collapse into 50,080 non-redundant sequences, which in turn are clustered into 8,504 major clusters with matches (E-value < 1 × 10^-5^) against HomoloGene. This clustering provides an estimate of the total number of transcribed protein coding genes identified in this study. This estimate is concordant with the total number of genes matched in the annotated *Tribolium *genome (9,303) at the same E-value cut-off (Table 2).

**Table 3:** Clustering using sequence matches to HomoloGene

E-value	Contigs		Singletons		Total	
	
	Query	Subject	Query	Subject	Query	Subject
1 × 10^-5^	17,160	11,504 (7,990)	1,807	2,934 (1,373)	18,967	12,464 (8,504)
1 × 10^-20^	11,032	8,084 (6,557)	845	1,145 (725)	11,877	8,524 (6,821)
1 × 10^-50^	5,711	4,767 (4,251)	183	190 (163)	5,894	4,846 (4,325)

The total numbers of non-redundant *Onthophagus *sequences with matches against the HomoloGene [54] database at the indicated E-value cut-offs. "Query" denotes the total number of *Onthophagus *sequences with matches against HomoloGene sequences, and "subject" denotes the total number of sequences from the HomoloGene database with matches against *Onthophagus*. The numbers of cases where the all *Onthophagus *sequences in a cluster have the *best *match to the same HomoloGene sequence are shown in parenthesis.

### Functional annotation

**Figure 4:**
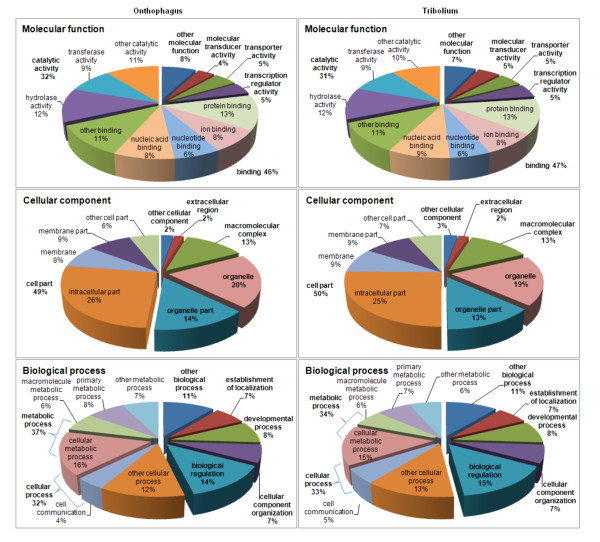
**GO categories**. GO annotations associated with 8,504 HomoloGene sequence groups. The distributions of the second and third levels of the GO term annotations of the sampled *O. taurus *sequences (left charts), were remarkably similar to those on the complete *T. castaneum *proteome (right charts).

We used sequence matches against the NCBI NR protein databases as a means of providing a first-pass annotation of putative function of the *O. taurus *non-redundant sequences. As expected the non-redundant sequences matched genes with a wide range of biological and biochemical processes (Additional file 6). Given that many of the non-redundant sequences derive from common genes, we turned to annotating the clustered sequences in order to gain a more accurate view of the range of biological processes represented by the expressed sequences. We examined the GO term annotations associated with the 8,504 HomoloGene groups that match the major clusters. Those clusters based on the best matches, i.e., major clusters, were searched for GO terms and the number of HomoloGene IDs was counted for each GO term. We used the annotated *T. castaneum *proteins as a reference for comparison. The distributions of the second and third level GO term annotations of the sampled *O. taurus *sequences were remarkably similar to those on the complete *T. castaneum *proteome (Figure 4). This indicates that the *O. taurus *sequences represent a broad sampling of biological processes. This interpretation was supported by examining the representation of annotated biochemical pathways. We mapped the major clusters to EC (Enzyme Commission) numbers and examined the distribution of these enzyme catalyzed reactions in a global metabolic map using iPath [55]. This revealed that the *O. taurus *expressed sequences included sequences encoding enzymes in all of the major categories of metabolic pathways including carbohydrate metabolism, lipid metabolism, energy metabolism, nucleotide metabolism, and amino acid metabolism (Additional file 7). Many of the core metabolic processes were well represented. For instance all of the steps in the TCA cycle, oxidative phosphorylation and fatty acid biosynthesis were found to be present. Thus the *Onthophagus *expressed sequences provide a good representation of genes with basic metabolic functions.

In addition to developing general comprehensive EST resources for the future study or horned beetle biology, our study also aimed to enrich existing pools of putative candidate genes specifically for the study of horn formation and plasticity in horned beetle development. We therefore examined whether our EST library contained contigs or singletons with matches to genes with GO terms believed to be relevant to horn formation or developmental plasticity (Table 4). For example, beetle horns develop from appendage anlagen that share many developmental properties with *Drosophila *imaginal discs [29], and we therefore searched for contigs matching genes with GO terms related to imaginal disc development and patterning. This effort yielded a rich set of candidate genes including prominent leg gap genes (e.g. *exd*, *dac*, *BarH1*) and several members of the *Notch*, *Wnt*, and *smoothened *signaling pathways. In addition to the likely importance of appendage patterning processes in horn development, horn formation is commonly sexually dimorphic [4], which is thought to be regulated by sex-specific differences in the endocrine regulation of horn induction and proliferation [8]. It was therefore of interest to us to search our EST library for contigs that match genes with GO terms related to sex determination as well as ecdysteroid and juvenile hormone (JH) metabolism and signaling. We identified several contigs that match cardinal sex determination and differentiation genes (e.g. *dsx*, *fru*), or have been implicated in JH or ecdysteroid function (e.g. *epoxide hydrolase 1*, *EcR*, *ftz-f1*, *usp*). Lastly, *Onthophagus *development is characterized by a highly variable degree of developmental plasticity in response to nutritional variation and we therefore examined our EST library for genes with GO terms related to the regulation of nutrition-mediated plasticity. Specifically, we searched our EST list for contigs that match genes related to DNA methylation (a mechanisms implicated in the nutritional control of caste differentiation in honey bees [56]) and insulin-signaling (suggested to play an important role in nutrient-mediated plasticity in horn development [57]). This effort identified the complete set of all three DNA methyltransferases (*dnmt*1-3) also reported from honey bees [56] as well as several prominent members of the insulin receptor signaling pathway (e.g. *InR*, *phosphatidylinositol 3-kinase*). These and additional examples are listed in Table 4. These results indicate that we greatly enriched the existing pools of candidate genes available for the study of horn formation and plasticity in horned beetle development.

**Table 4:** Candidate developmental genes

Sequence ID	Accession	E-value	Description	GO
contig19562	NP_001034532.1	9.00E-99	brachyury	1
FQTIJGT01DV55X	NP_001034527.2	2.00E-53	Kruppel	1
contig27749	XP_970831.2	1.00E-23	PREDICTED: similar to fibroblast growth factor receptor	1
contig14082	XP_001602830.1	1.00E-137	PREDICTED: similar to epidermal growth factor receptor	1, 2, 4, 21
contig13096	XP_001654153.1	1.00E-108	decapentaplegic	1, 4, 21
contig18654	XP_975017.2	0	PREDICTED: similar to ets	1, 8
contig18756	BAD00045.1	0	armadillo protein	1, 9, 16
contig13865	XP_970668.2	1.00E-58	PREDICTED: similar to Homeobox protein cut	1, 10
contig04562	NP_001107765.1	3.00E-45	hairy	1, 10, 21
FQTIJGT02F4ASF	NP_001034490.1	4.00E-23	pangolin	1, 15
contig17509	XP_968516.2	0	PREDICTED: similar to par-1 CG8201-PA	1, 15
contig00028	XP_967537.1	1.00E-180	PREDICTED: similar to COUP-TF/Svp nuclear hormone receptor	1, 17
contig14224	XP_970678.1	1.00E-156	PREDICTED: similar to thickveins CG14026-PA	1, 21
contig08201	XP_974235.1	6.00E-37	PREDICTED: similar to DNA cytosine-5 methyltransferase	3
FQTIJGT01BFBE3	XP_974854.1	8.00E-69	PREDICTED: similar to cornichon protein, putative	2
contig19544	XP_966833.1	0	PREDICTED: similar to extracellular signal-regulated kinase	2
contig25027	XP_968594.2	1.00E-115	PREDICTED: similar to Ecdysone-induced protein 63E CG10579-PK	5
contig04278	XP_396527.3	1.00E-176	PREDICTED: similar to Ecdysone-induced protein 78C CG18023-PA,	5, 17
contig32340	XP_001847468.1	5.00E-89	ras	5
FQTIJGT01E6R2M	NP_001116500.1	2.00E-12	matrix metalloproteinase 1 isoform 2	5, 9
contig20604	XP_001663781.1	3.00E-17	phosphatidylinositol 3-kinase regulatory subunit	6
contig33698	XP_001952079.1	1.00E-19	PREDICTED: similar to insulin receptor	6
contig26215	XP_974994.1	4E-70	PREDICTED: similar to Phosphatidylinositol-3,4,5-trisphosphate 3-phosphatase and dual-specificity protein phosphatase PTEN	6, 8
contig36880	NP_001128399.1	2.00E-14	epoxide hydrolase 1	7
contig20325	NP_001034501.1	0	extradenticle	4
contig04888	XP_969771.2	6.00E-45	PREDICTED: dachshund	4
contig29998	XP_001944887.1	9.00E-75	PREDICTED: similar to BarH1 CG5529-PA	4
contig07732	XP_969484.2	3.00E-30	PREDICTED: similar to LIM homeobox 1b	4
contig01196	NP_001034489.1	1.00E-152	homothorax	4, 18
FQTIJGT02HBUI7	NP_001107853.1	1.00E-38	Notch	11
contig26747	XP_975449.2	4E-20	PREDICTED: similar to FAS-associated factor 1, putative	8
contig14846	NP_001034510.1	1E-126	transcription factor deformed	8, 18
contig03161	AAO16241.1	3.00E-86	effector caspase; Sl-caspase-1	9
mira_c460	XP_001810562.1	3.00E-31	PREDICTED: similar to caspase	9
contig02560	XP_966617.2	2.00E-77	PREDICTED: similar to E74	9
contig02035	XP_967068.2	2.00E-19	PREDICTED: similar to NAD-dependent deacetylase sirtuin-1	9, 12
contig22079	XP_970822.2	0	PREDICTED: similar to Darkener of apricot CG33553-PG	9, 19
contig20962	NP_001107840.1	2.00E-67	Dicer-2	12
contig04521	XP_971295.2	0	PREDICTED: Argonaute-1	12
contig35987	XP_624270.2	1.00E-109	PREDICTED: similar to brahma CG5942-PA, isoform A, partial	12
contig03878	XP_975376.1	1.00E-70	PREDICTED: similar to Headcase protein	12
contig04464	XP_966633.1	0	PREDICTED: similar to histone deacetylase	12
contig35227	NP_001107838.1	5.00E-40	aristaless	10
contig36981	XP_001814382.1	1.00E-156	PREDICTED: similar to fringe CG10580-PA	10, 11
contig09571	XP_975412.2	2.00E-53	PREDICTED: similar to suppressor of fused	13
contig04709	XP_975408.1	0	PREDICTED: similar to supernumerary limbs CG3412-PA	13, 15
FQTIJGT02G66FW	EEB10664.1	6.00E-37	Antennapedia, putative	18
contig06860	AAK96031.1	3.00E-82	homeodomain transcription factor Prothoraxless	18
contig04152	NP_001107807.1	0	maxillopedia	18
contig08220	XP_971065.1	5.00E-96	PREDICTED: similar to rotated abdomen CG6097-PA	18
contig05318	NP_001034497.1	1.00E-107	ultrabithorax	18
contig02060	XP_971671.2	4.00E-44	PREDICTED: similar to fruitless	20, 19
contig25669	XP_001807448.1	1.00E-58	PREDICTED: similar to BmDSX-F	19
contig14519	XP_971676.1	2.00E-70	PREDICTED: similar to iroquois-class homeodomain protein irx	14
contig15982	XP_968422.1	1.00E-117	PREDICTED: similar to cadherin	14, 21
contig22068	NP_001127850.1	2.00E-88	smoothened	14, 21
contig31931	NP_001107650.1	2.00E-94	ecdysone receptor isoform A	17
FQTIJGT02HPUTA	XP_001845875.1	6.00E-67	nuclear hormone receptor FTZ-F1 beta	17, 22
FQTIJGT02GV771	XP_971362.2	7.00E-18	PREDICTED: similar to ecdysone inducible protein 75	17
contig03369	CAH69897.1	1.00E-162	retinoid X receptor	17
contig04903	NP_001107813.1	1.00E-122	glass bottom boat protein	21
contig05941	XP_971286.2	0	PREDICTED: similar to mothers against dpp protein	21
contig07923	EEB19343.1	8.00E-19	porcupine, putative	16
contig01101	XP_968118.1	5.00E-45	PREDICTED: similar to frizzled	16
contig08319	XP_623523.1	4.00E-75	PREDICTED: similar to frizzled 7	16
contig03739	XP_974963.2	1.00E-172	PREDICTED: similar to jnk	16
contig13446	XP_973551.1	1.00E-150	PREDICTED: similar to legless CG2041-PA	16
FQTIJGT02I9RJB	XP_969261.1	3.00E-13	PREDICTED: similar to Wnt11 protein	16
contig29078	XP_968055.2	1.00E-77	PREDICTED: similar to Wnt6	16
contig19316	XP_974084.1	1.00E-70	PREDICTED: similar to wntless CG6210-PB	16
contig19245	XP_001847858.1	1.00E-168	wingless protein	16

Examples of contigs representing genes putatively involved in *Onthophagus *development. contig/singleton ID, accession number from NCBI NR dataset, E-value, and gene description are shown. Specific GO terms shown here are: 1. cell fate determination, 2. epidermal growth factor receptor signaling pathway, 3. DNA methylation, 4. leg disc pattern formation, 5. instar larval or pupal development, 6. insulin receptor signaling pathway, 7. juvenile hormone metabolic process, 8. positive regulation of programmed cell death, 9. programmed cell death, 10. regulation of Notch signaling pathway, 11. Notch signaling pathway, 12. regulation of gene expression, epigenetic, 13. regulation of smoothened signaling pathway, 14. smoothened signaling pathway, 15. regulation of Wnt receptor signaling pathway, 16. Wnt receptor signaling pathway, 17. steroid hormone receptor activity, 18. segment specification, 19. sex differentiation, 20. sex determination, 21. wing disc pattern formation, 22. response to ecdysone.

### Sequence polymorphisms

Our libraries sampled a total of 64 haploid genomes (32 diploid individuals) from a laboratory culture established from wild caught animals, which allowed us to begin to explore sequence variation in *O. taurus*. After merging contigs in BCCs, all contigs were used for sequence variant analysis. GigaBayes [58] identified 164,537 SNPs and 344,632 indels. After removing indel calls in homopolymer regions, 80,732 indels remained. Additional file 8 shows histograms of our confidence in identified sequence variants (based on the "probability" value calculated for each SNP by GigaBayes). We focused subsequent analyses on only high quality sequence variants: those with at least 5 reads and a GigaBayes probability value of at least 0.9. Additional file 9 shows sequence changes of 92,979 and 25,496 final SNPs and indels. Transitions - A-G and T-C mutations - were more common than transversions. Furthermore, insertions and deletions more commonly affected A and T than C and G.

Across all contigs, the average SNP frequency was 0.00567 SNP/bp, or approximately 1 SNP for every 176 base pairs (for insertions and deletions, mean = 0.00197 indels/bp). The number of SNPs in a contig was positively related to both contig length and number of reads in a standard least squares linear model containing both transformed variables (Table 5). The number of insertions and deletions in a contig was also related to contig length and read number in a separate model (Table 5). In subsequent analyses, we considered residual SNP and indel frequency (observed - expected, given contig length and read number), which allowed us to estimate genetic variation while correcting for variation among contigs in read depth and length.

**Table 5:** Effect of contig length and coverage on detection of SNPs and Indels

	Total detected SNPs			Total Detected Indels		
	*Estimate*	***F***_***1,38047***_	*P*	*Estimate*	***F***_***1,38047***_	*P*
Contig Length	1.73	4371	0.0000	0.128	138.8	<0.0001
Number Reads	0.96	5417	0.0000	0.313	3304	0.0000

Both contig length (transformed) and number of reads (transformed) were related to total detected SNPs and total detected indels in standard least squares linear models. Residuals from these models were used in subsequent analyses to estimate levels of genetic variation in a contig controlling for sampling differences between contigs.

**Figure 5:**
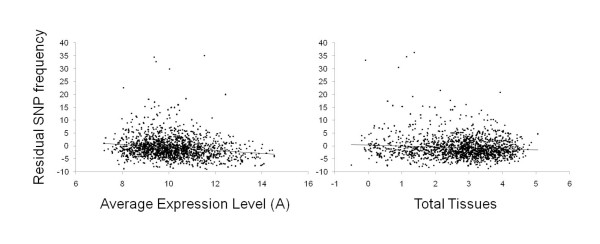
**Gene expression patterns are correlated with patterns of genetic variation**. Residual SNP frequency (number of SNPs in a contig controlling for contig length and read number) was negatively related to overall expression levels ("A") and the number of tissues (head horn epidermis, thoracic horn epidermis, legs and central brain) in which differential expression was detected in a previous microarray study (N = 48 arrays, reported in Snell-Rood et al. 2010). Statistics are presented in Table 6.

We aligned residual SNP and indel frequency with a previously reported expression data set [46]. Expression patterns were measured in females, large horned males and small sneaker males in three epidermal tissues (head horn, thoracic horn and legs) relative to abdominal epidermis and in central brain tissue, relative to ganglionic brain tissue. These array data (N = 48 arrays) were used to estimate overall expression levels of a contig ("A"), total tissues in which differential expression was detected, bias between male morphs and sexes (across all tissues, see [46]). We found that SNP and indel frequency were negatively related to the overall expression level of a gene and the number of tissues (the inverse of tissue specificity) in which the gene was expressed, in a standard least squares linear model that also controlled for morph-biased and sex-biased expression (Table 6, Figure 5).

**Table 6:** Correlations between patterns of gene expression and estimated levels of variation

	Residual SNP frequency			Residual Indel Frequency		
	*Estimate*	***F***_***1,1538***_	*P*	*Estimate*	***F***_***1,1538***_	*P*
Average Expression	-0.565	46.5	<0.0001	-0.083	8.19	0.004
Number of Tissues	-0.364	13.1	0.0003	-0.063	3.23	0.07
Morph-biased Exp.	-0.127	0.42	0.52	-0.032	0.22	0.64
Sex-biased Exp.	0.108	0.30	0.58	-0.009	0.02	0.89

Shown are results from standard least square linear models relating measures of gene expression from a previous experiment - average expression levels (A), sex-biased gene expression, alternate mating morph-biased gene expression, and expression detected across up to four different tissue types - to estimates of genetic variation (see Table 5).

We used Blast2Go to identify GO terms enriched in subsets of genes with more or less variation than expected. The most variable genes (the top 5% of genes in terms of residual SNP frequency) were enriched for three GO terms, including actin binding and cytoskeletal protein binding (Additional file 10, Sheet "top 5% SNP variation"). The least variable genes (the lowest 5% of genes in terms of residual SNP frequency) were enriched for 61 GO terms, including many processes related to metabolism, development, intracellular functions, cell cycle, nucleic acid binding, anatomical structures, and life history (Additional file 10, Sheet "lowest 5% SNP variation").

## Discussion

Our results build on a growing literature that shows that 454 pyrosequencing can be a powerful tool for expanding genomic resources for emerging model systems (e.g. [59,60]. The major shortcoming of such high-throughput sequencing - short reads that can be difficult to assemble - are being overcome by advances in the sequencing process itself and novel bioinformatic approaches explored in studies such as this. Here we review our findings, the novelty of our approach, and highlight some of the specific tools this sequencing effort brings to the *Onthophagus *system.

### Sampling the *Onthophagus *transcriptome

As in most high-throughput sequencing projects, in one 454 run, we generated a massive body of sequence information: almost 600 millions base pairs spread over 1.4 million cleaned reads (Table 1). This has vastly expanded our existing set of *Onthopagus *ESTs that was previously generated by Sanger sequencing [30], with 93% of the 454 sequences (46,891) failing to match the Sanger sequences (E-value cut-off = 1 × 10^-50^). Nevertheless the Sanger ESTs are not entirely redundant, as 9.6% of the Sanger sequences failed to match the 454 sequences (E-value cut-off = 1 × 10^-50^). Using a series of analyses, these reads were assembled into contigs and clusters that represent approximately 8,500 known genes (4,000 - 14,000 depending on the database used and the stringency for the match; see Table 2, 3). Our analyses provide several lines of evidence that suggest we can be confident in our assembly.

First, the majority of our sequences significantly align with known protein sequences, in particular those of *Tribolium*, the only beetle genome currently sequenced. Specifically, 52% of contigs and singletons, and 59% of contigs align with known sequences from one of three databases (E-value < 1 × 10^-5^; Figure 1). The majority of these alignments (80%) agree between all three databases queried (see Table 2). A small number of genes (<4% of sequences) matched a protein in only one of the databases; these genes likely represent instances where the *Tribolium *genome is incompletely annotated or assembled or where genes are transcribed in *Onthophagus*, but not in *Tribolium *(see Figure 1).

While the majority of our sequences aligned with known sequences, 41-48% of our sequences did not match known proteins or the *Tribolium *genome. Our analyses suggest that many of these sequences represent contigs with short or very few reads, or reads that cover non-coding sequence (Additional file 3). However, at least 20% of these genes are of similar or greater quality (average read length and proportion coding sequence) of sequences with significant alignments, and may include genes that are novel or highly divergent between *O. taurus *and *Tribolium*. This is not surprising given that *Onthophagus *and *Tribolium *shared a common ancestor over 150 million years ago [61]. These divergent genes may be fruitful for future research given that they may represent novel genes, or genes under strong selection for new functions.

A second line of evidence that generates confidence in our results is a survey of the taxonomic distribution of best sequence matches. Specifically, of contigs with significant matches to the NCBI NR database, over 8,000 (40%) were classified to *Tribolium*, and 18,500 (87%) to Arthropoda. A relatively small proportion was classified to taxa outside of insects. For instance, less than one percent of genes were classified to bacteria. Given these beetles feed on dung and have a diverse associated gut flora [62,63], the non-arthropod-classified sequences could represent gut contamination of symbionts or partially digested plant products. We sought to minimize such contamination by sampling only the head and thorax of individual beetles, but presumably, contaminants could be present in the foregut or structures specialized to house symbionts. The fact that non-arthropod classified sequences are dominated by singletons, while the arthropod-classified sequences are dominated by contigs (Figure 2), supports the interpretation of minor contamination by naturally associated plant parts and bacteria.

Our Gene Ontology classification also lends significant support to our assembly of the *Onthophagus taurus *transcriptome. Comparing *Onthophagus *to *Tribolium*, our genes sample roughly the same proportion of gene classes for classifications of molecular function, cellular component, and biological process (Figure 4). For instance, in *Tribolium*, 15, 9 and 8 percent of genes are involved in "biological regulation," "developmental process" and "establishment of localization," respectively, while in *Onthophagus*, the corresponding percentages are 14, 8, and 7. The largest differences are still modest and fall within the "metabolic process" category (39% of *Onthophagus *genes fall into this category, versus 32% of *Tribolium *genes); which could prove to be an interesting consequence of the differences in diet between the two species.

By comparing our assembled sequences to existing databases, our analyses suggest that we have sampled about half of the *Onthophagus *transcriptome. For instance, around 20,000 contigs and singletons match to approximately 10,000 separate genes (depending on the database used; at an E-value of 1 × 10^-5^; see Table 2, 3). Based on the *Drosophila *genome, we can estimate that *Onthophagus *may possess around 20,000 genes; thus, we have sampled about half of the genes present in the genome. If we assume that the 4,000 higher quality, but highly diverged genes discussed above, which did not align to known genes, match to about 2,000 additional, independent genes (as in Table 2, 3), then we may have sampled about 60% of the transcriptome. Regardless, this dataset represents a rich resource for future work on the system.

### Bioinformatic Approaches

Next generation sequencing and the *de novo *assembly of transcribed sequences is increasingly being used to characterize the transcriptomes of non-model organisms for which a whole-genome sequence is not yet available [64]. The absence of a reference genome sequence makes the assembly of these sequences particularly challenging. Because 454 sequencing results in sequence reads that are generally shorter than a given gene, assembly relies on generating a series of overlapping reads. However, any sequence variants among the reads, for instance due to genetic variation between individuals, sequencing of paralogs of a gene, or alternative splicing, can make assembly difficult. Furthermore, overlapping reads may be assembled for separate components of a gene, resulting in multiple contigs representing one gene. We used several complementary bioinformatic approaches to overcome the limitations of short read lengths.

We used a clustering approach against the HomoloGene database to determine whether our non-redundant sequences (contigs and singletons) derived from the same gene within the genome. Similar approaches have been demonstrated to be effective in clustering contigs of transcribed sequences in the absence of a reference genome [65-67]. This method allowed us to identify the highest confidence set of non-redundant protein-coding sequences in our dataset. For instance, with an E value < 1 × 10^-5^, 18,976 *Onthophagus *sequences matched sequences from 12,464 HomoloGene groups (Table 3). This method was concordant with our more general approach to identify the number of genes sampled, where we tested for sequence matches across several databases, including the *Tribolium *genome (Table 2).

In the absence of a reference genome sequence it is very difficult to identify sequences that derive from the same gene but fail to assemble due to sequence polymorphisms or alternative splicing. This is particularly true in the absence of informative similarities to sequences from related species. One approach to this problem has been to remove a sub-set of redundant contigs without resolving the relationship between the sequences [68]. Here we used a novel analysis of "broken reads," individual sequence reads that were placed in two or more contigs during assembly. This approach identifies groups of sequences connected through multiple broken reads ("bi-connected components") and are thus biologically linked, but have failed to assemble due to sequence polymorphisms, alternative splicing or gene duplication (see Figure 3). To our knowledge this approach is unique in being able to cluster such sequences in the absence of a reference genome. We then used sequence similarity amongst the sequences within bi-connected components to infer the most likely physical origin of the connected sequences. For instance, when linked contigs shared at least 95% sequence identity, they were assumed to be divergent alleles and the sequences were merged. Of the sets of linked contigs that fit this criterion, 99% (1,547/1,565) matched to the same protein in the NCBI NR database, suggesting our assumptions were correct. Linked contigs with less sequence similarity were classified as either putative duplicates (85-95% similarity) or alternatively spliced transcripts (<85% sequence similarity), both of which were supported by comparing our classifications to existing gene models. It is important to emphasize that while the clustering of bi-connected components provides very strong evidence that the sequences derive from either alleles, alternatively spliced transcripts, or gene families, the classification based on sequence similarity is not definitive. The definitive resolution of gene structures must ultimately rely on genome and/or full length cDNA sequencing. Despite this caveat the clustering based on broken reads is an effective means of grouping related sequences in the absence of a reference genome.

Our preliminary SNP analyses lend further confidence in the classification of genes as alternative alleles. First, we found that transitions were more common than transversions (Additional file 9), as commonly reported in studies that consider patterns of genetic variation [69-72]. Second, we used previously reported microarray data [30,46] to test whether the SNPs we identified were related to patterns of gene expression in manners consistent with other studies. We found significant negative relationships between SNP frequency and overall levels of gene expression, consistent with the commonly reported relationship between gene expression and gene conservation [73,74]. We also found a negative relationship between SNP frequency and the number tissues in which a gene was significantly expressed, which is reminiscent of the positive relationship between tissue-specific gene expression and sequence divergence due to pleiotropic constraints [75,76].

Overall, our use of multiple, complementary bioinformatic approaches allowed us to glean a large amount of information from one 454 run. We are now primed for a range of studies on the *Onthophagus *system, some of which we highlight below. Furthermore, this analysis pipeline will allow similar resources to be developed for a range of emerging model systems.

### Tools for Future Studies of this Model System

The resources generated in the present study provide an expansive toolbox for advancing current, and enabling future research efforts in horned beetles. Here, we highlight three particularly interesting avenues for future inquiry.

#### The Origin of a Novel Trait: Horn development

Horned beetles in general, and *Onthophagus *beetles in particular, are becoming attractive models for studying the origin and diversification of novel traits, specifically horns [21]. Horns lack obvious homology to other traits in insects or non-insect arthropods, yet develop at least in part similar to more traditional appendages such as legs and mouthparts. Thus, beetle horns offer an interesting opportunity to study how evolutionary changes in the interactions between ancestral developmental-genetic mechanisms may enable the origin of novel features. Earlier studies have begun to implicate several developmental pathways in the regulation of horn development using immunohistochemical analysis of candidate genes (e.g. limb patterning [28,29], programmed cell death [44]), quantitative PCR (e.g. insulin signaling [57]), hormone manipulations (e.g. juvenile hormone metabolism [9]) and most recently RNAinterference [43]. In each case, analysis of candidate genes was limited to very few or one gene candidate. In the present study we substantially increase the number of candidate genes now available to investigate the role of these pathways in horn development and evolution (Table 4). Furthermore, we provide a substantial number of candidate genes for the investigation of developmental pathways previously *inaccessible *for study, yet hypothesized to play a potentially significant role in the origin and diversification of beetle horns and horned beetles, such as the notch, Wnt, and EGFR signaling pathways [77]. In so doing the present study also contributes important resources for moving beyond the examination of single candidate genes and toward examining interactions between developmental pathways and within and between gene networks.

#### Phenotypic Plasticity

The biology of horned beetles is characterized by a remarkable degree of phenotypic plasticity - a single genotype's ability to adjust phenotype expression to changes in environmental conditions[78]. In horned beetles, such plastic responses involve behavioral traits (e.g. fighting vs. sneaking reproductive tactics in males [79]), parental investment [25,26], physiological changes (timing of metamorphosis [24], thermoregulation [80]) and morphology (horns [27], testes investment [81]). However, the developmental genetic basis of this plasticity is largely unknown. Here, our 454 run has generated a rich set of tools for future inquiry into the genetic underpinnings of phenotypic plasticity. For instance, the insulin signaling pathway has been suggested to play an important role in mediating the plastic switch between fighter and sneaker tactics in horned beetles [57]. Our sequencing effort identified many important genes in this pathway, including *chico*, *FOXO*, *insulin receptor*, and *melted*.

Similarly, DNA methylation is another important candidate pathway for nutrition-induced phenotypic plasticity [82]. The importance of DNA methylation in insects has been of interest in recent years, following the discovery that a complete methylation machinery - while absent in *Drosophila *- is present in the Hymenoptera [83], shows significant variation across species [84], and appears to play an important role in nutrition-induced caste determination in honeybees [56,85]. The present study has identified the complete set of all three DNA methyltransferases (*dnmt*1-3) in *Onthophagus taurus*, including the *de novo *methyltransferase (*dnmt*3) and the maintenance methyltransferase (*dnmt*1), setting the stage for future studies into the role of DNA methylation in horned beetle plasticity

#### Population Genetics and Patterns of Genetic Variation

Our SNP analyses identified overall levels of genetic variation comparable to 454 studies of other animals sampled from wild populations [70,71], but considerably more relative to domestic or lab strains of animals [59,86] and considerably less relative to plants, even domestic varieties [69,87]. This SNP dataset will serve as a powerful resource in future studies of genetic variation. We can now easily genotype individuals and sample standing levels of genetic variation. Having SNPs associated with this assembly also primes us for more powerful analyses of how gene expression patterns affect genetic variation, for instance due to relaxed selection on morph- or environment-specific genes [88].

Our SNP dataset also brings us closer to identify patterns of natural selection on the *Onthophagus *genome, and determining which genes are under strong positive or purifying selection. Such analyses will be facilitated by sequencing other species in the genus (and the calculation of dN/dS). Until then, we can get hints at classes of genes under strong or weakened selection, based on our gene ontology enrichment analysis of more or less variable genes. For instance, very few GO categories were associated with highly variable genes (actin binding, cytoskeletal protein binding; Additional file 10). This could be because these variable genes are more divergent and have no or incomplete annotation. Indeed, of these 1900 genes (5% of 38,000 contigs), 47.4% were annotated in the low variation group and 33.2% were annotated in the high variation group. In contrast, the least variable genes were enriched for over 60 GO terms (Additional file 10). Scanning this list reveals many processes that are likely under purifying or positive selection, including metabolism (GO terms: metabolic process, primary metabolic process, macromolecule metabolism, etc.), development (developmental process, multicellular organismal development), cell cycle (cell cycle, cell death), morphology (anatomical structure development and morphogenesis, nucleic acid binding (nucleotide binding, RNA binding), and life history (death, reproduction). While these processes may be biologically relevant and possibly indicative of the origin and rapid diversification of novel traits in this lineage (horns), it is important to treat these lists with caution as such enrichment analyses can be confounded by nested gene ontology categories [89]. Regardless, this analysis yields genes and developmental processes that may prove interesting for future study in this system.

## Conclusions

This study sampled sequences from approximately half of genes expressed in the horned beetle *Onthophagus taurus. *This greatly advances our knowledge of the *Onthophagus *transcriptome and paves the way for future molecular genetic studies of horn evolution and development.

## Methods

### Sample preparation and sequencing

Beetles used in this study were reared as described previously [29]. To avoid possible contamination of the gut fauna, we used head and three thoracic segments from our larval samples. Instead of dissecting head and thoracic segments from pupae, we used the whole body. Late pupae were transferred to a clean, humid chamber before emergence and adults were not fed with any cow dung to avoid possible contamination from the food source. To enrich the pool of expressed genes with various classes of transcripts, we included all the major stages of postembryonic dung beetle development: mid 3rd instar larva (5 days after molt), late 3rd instar larva (11 days after molt), early and late prepupa, pupa within 24 hrs after pupation, between 36 and 48 hrs after pupation, mid pupa (7 and 9 days after pupation), late pupa (12 days after pupation), and adults 4 days after molt. Each stage includes two males and two females except for pupa day 7 (one female) and day 9 (one male). In total, we used 32 animals to extract total RNA. Total RNA was isolated as described in [30]. RNA quantity and quality were tested using an Agilent Bioanalyzer 2100. Total RNA was then sent to the Center for Genomics and Bioinformatics at Indiana University, Bloomington (IU CGB), which prepared a normalized transcriptome (cDNA) library optimized for Roche 454 GS-FLX Titanium sequencing using custom methods (K. Mockaitis, unpublished, available upon request). This library was sequenced using the GS-FLX Titanium process on a full PicoTitre plate, according to the manufacturer.

### Sequence assembly

**Figure 6:**
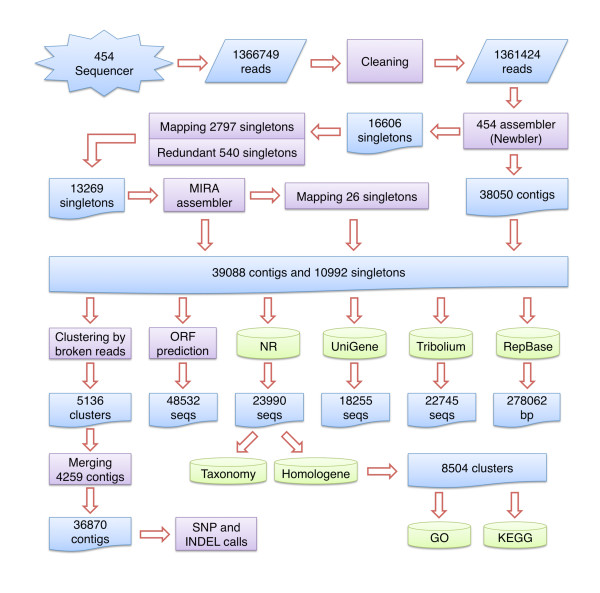
**Flow diagram of sequence assembly and annotation**. Flow diagram illustration the steps involved in the sequence analysis. Computational steps are indicated by purple boxes, sequences are indicated by blue boxes, and analysis with respect to sequence or annotation databases are indicated by green cylinders.

The flow of information in the sequence data analyses is summarized in Figure 6. Sequence reads were cleaned using an in-house package [90] The sequences were assembled as follows. First, sequences were assembled using the 454 Newbler assembler [48] with a minimum overlapping length of 40 bp and a minimum percent identity of 90%. This assembled 38,050 contigs (22 Mbp) leaving 16,606 singletons (5.8 Mbp) and discarding 48,409 sequences due to them being too short, chimeric, or repetitive. Second, putative false negative assemblies were identified by Blast aligning the singletons against the contigs. This identified 2,797 singletons that aligned with 2,351 contigs (≥95% percent identity and ≤10 bp unaligned bases at either end). These singletons were merged with the respective contigs. Third, redundant singletons were identified by Blast aligning remaining singletons *inter se*. This identified 540 redundant singletons (≥95% percent identity and ≤10 bp unaligned bases at either end) that were discarded. Fourth, the remaining unassembled singletons were assembled using the MIRA assembler [49]. This assembled 2,251 singletons into 1,038 contigs (444 Kbp). Finally, the remaining singletons were Blast aligned to the Mira contigs and 26 singletons were merged to 25 contigs (≥95% percent identity and ≤10 bp unaligned bases at either end). This procedure produced 39,088 contigs (23 Mbp) and 10,992 singletons (3.7 Mbp) which are referred to here as the *O. taurus *non-redundant sequences.

### Sequence alignments and analysis

Repeats, transposons, small RNAs and low complexity regions were identified using RepeatMasker [50] in conjunction with the RepBase [51] database for *Drosophila*. *O. taurus *sequences were aligned to the following public sequence databases using BlastX and tBlastX (-F F -e 1e-5): NCBI *T. castaneum *UniGene #12 [91], *T. castaneum *genome v3.0 [92], *T. castaneum *annotated proteins v3.0 [92], and NCBI HomoloGene build 64 [54]. Open reading frames (ORFs) were predicted using ORF finder [93] and ORFpredictor [94]. The taxonomical analysis of sequence matches was performed using MEGAN [53]. Gene Ontology analysis was performed using Blast2Go [95]. The analysis of metabolic pathways was performed by first using Blast sequence matches against HomoloGene [54] to assign Enzyme Commission (EC) numbers, and then using iPath [55] to visualize metabolic pathways.

### Clustering

Two different approaches were used to cluster non-redundant sequences. In the first approach broken reads - reads that were split and assembled in two or more contigs by Newbler - were used to group contigs. Using a custom script (script available at: https://www.sourceforge.net/projects/snp454), broken reads were used to construct graphs; where nodes represented contigs and edges represented broken reads, and contigs forming connected components (CC) and bi-connected components (BCC, subgraphs that are not split if any one edge is removed) were identified. Briefly, the script was fed Newbler assemblies in ACE format and performed the following steps: (i) identify broken reads based on read names, (ii) construct graph using Pearl Graph Module [96], (iii) identify CC and BCC using Pearl Graph Module function, (iv) flag each BCC as putatively allelic, duplicated, or alternative splicing according to the criteria described below.

The contigs connected in BCCs are likely to represent allelic variants, duplicated genes, duplicated exons, alternative splicing, or a combination of the aforementioned. It is to be expected that contigs that failed to assemble due to allelic variation will share higher sequence similarity than duplicated genes/exons, and that alternatively spliced exons will share the lowest sequence similarity. In order to explore whether we could distinguish between these classes based on sequence similarity alone, we analyzed the sequence similarity between recently duplicated genes [97], and between alternatively spliced exons in the *D. melanogaster *genome (FlyBase FB2010_07). Recently duplicated genes (e.g. *Nuclear transport factor-2 *and *Sperm-specific dynein intermediate chain*) can share regions of up to 95-100% sequence identity. Given that this exceeds the sequence similarity cut-off commonly used for the assembly of 454 sequences [48,49], it is not possible to definitively distinguish between BCCs representing allelic variants from those representing duplicated genes. However, an earlier scan for segments duplicated in the *D. melanogaster *genome identified 82 groups of duplicated sequence (average length 3.7 kb) that share 95% or greater sequence identity [98], indicating that such cases are rare. On the other hand 97% of alternatively spliced genes in *Drosophila *do not have pairs of exons with Blastn matches with E-value < 1 × 10^-5 ^(excluding overlapping exons). Thus, while most alternatively spliced exons fall below this cut-off, there are exceptions and it is not possible to definitively distinguish between BCCs representing alternative splicing from those representing duplicated genes/exons or allelic variation, based on sequence similarity alone. Having established that sequence similarity performs reasonably well in classifying alleles, duplicated genes and alternatively spiced exons; we performed *inter se *Blastn sequence alignments among the contigs within BCCs, and classified them as follows. First, highly similar sequences were flagged as putative allelic sequences if they met the following criteria: at least 50 bp of at least 95% sequence identity, flanked on either side by no more than 10 nt of less than 95% sequence identity (single stranded overhangs of >10 nt were permitted). Second, pairs of contigs not meeting the first criteria but having Blastn matches of E-value < 1 × 10^-5 ^were flagged as putatively representing duplicated genes. Finally, BCCs with contigs failing to meet the first two criteria were flagged as putatively representing alternatively spliced transcripts.

The second approach to cluster non-redundant sequences utilized sequence matches to the HomoloGene database of groups of homologous sequences from sequenced genomes [54]. The non-redundant sequences were assigned HomoloGene IDs based on the best Blast matches to the database (minimum cut off = E-value < 1 × 10^-5^). Clusters of *O. taurus *non-redundant sequences that all shared the *best *Blast match to the *same *HomoloGene group were defined as "major clusters".

### Sequence Variants

Sequence variant call programs suffer from the fact that when assembling contigs, the Newbler algorithm introduces gaps instead of substitutions in alignments between reads. To overcome this problem, we realigned sequences within contigs using a custom script (Script available at: https://www.sourceforge.net/projects/snp454). This script was fed Newbler alignments in ACE format and performed following steps: (i) extracted pairwise alignments, (ii) removed homopolymeric gaps from pairwise alignments, and (iii) ran MosaikAssemble [99] to generate multiple sequence alignments. These multiple sequence alignments were then fed to GigaBayes [58] to predict sequence variants. The sequence variants predicted by GigaBayes were filtered out for high confident sites if indel variants occur in homopolymer regions, read coverage is less than 5 or greater than 100 and the probability of sequence variants is less than 0.9.

To analyze patterns of SNP variation, we first calculated a "residual" SNP number from a standard least squares linear model that controlled for read length and number of reads (both factors were first log transformed and treated as fixed effects in the model). Previous studies have acknowledged the importance of controlling for both factors when estimating genetic variation from 454 data [72], but we feel this analysis improves on previous metrics. By using a residual calculated from a predicted value, we can control for the fact that a SNP reading of "0," could be due to low genetic variation, *or *low power due to short read lengths. We performed several exploratory analyzes of SNP variation. First, we used past microarray data to test if genetic variation was related to gene expression patterns. Our microarray data [46] were based on the past cDNA EST assembly [30] that was incorporated into the current assembly. For any microarray construct that matched more than one contig in the 454 assembly we averaged the residual SNP frequency. Expression data are described in detail in [46]. Briefly, gene expression was measured in first day pupae of females, large, horned male and small, sneaker males in the head horn epidermis, thoracic horn epidermis and legs relative to abdominal epidermis and in the central brain relative to ganglionic neural tissue (N = 48 total arrays). We focused on four measures of gene expression: total expression level ("A"), total tissue types (out of 4) in which differential expression was detected, bias in gene expression between male morphs and between males and females (averaged over all tissues). We recognize that the relationship between tissue specificity and SNP frequency could be confounded by our method of detecting SNPs. Specifically, a highly expressed, tissue-specific gene may be detected only in one or two individuals (that are currently expressing this gene), thus decreasing the probability of detecting SNPs in that gene. However, this prediction is opposite that predicted (and found) in our data; that tissue-specific genes are more variable. In our second set of analyses, we were interested in whether genes with the greatest or least amount of variation were enriched for any GO terms. We performed an enrichment analysis using Fisher's Exact Test implemented in Blast2Go [100].

## Authors' contributions

J-HC, TK, ES-R, APM, and JA designed the study. TK developed cDNA libraries. J-HC, HT and Y-IY performed computational analyses. ES-R performed SNP-expression analyses. J-HC, TK, ES-R, APM and JA wrote the paper. All authors read and approved the final manuscript.

## Supplementary Material

Additional file 1 **Assembled sequences and singletons**. A text file containing 39,088 contig and 10,992 singleton sequences in FASTA format. Click here for file

Additional file 2 ***Onthophagus taurus *repeated sequences**. A table containing a summary of repeated sequences by RepeatMasker. Click here for file

Additional file 3 **Comparison of read quality between sequences with and without database hits**. "Hit" refers to contigs with significant (e value < 10^-5^) match against the Tribolium genome and protein databases and/or the NCBI NR database (N = 21847 total). "No Hit" refers to sequences with no significant database match (N = 16203). Shown are histograms for contig length, the proportion of a contig that represents coding sequence, and total read number for a contig. Click here for file

Additional file 4 **BCCs**. An excel file showing BCCs with NR protein database support. Click here for file

Additional file 5 **HomoloGene clusters**. An excel file showing HomoloGene clusters. Click here for file

Additional file 6 **Blast matches of non-redundant sequences to NCBI NR proteins**. An excel file showing contigs and singletons that match to NCBI NR proteins. Click here for file

Additional File 7 **Metabolic pathways map**. A metabolic pathway map showing the steps represented by *Onthophagus *sequences (thick lines). Click here for file

Additional file 8 **Confidence distribution of sequence variants**. A figure showing frequency histograms of the confidence scores of sequence variants. "Major allele" refers to the more common sequence variant, while "minor allele" refers to the rarer sequence variant. Click here for file

Additional file 9 **Sequence changes of SNPs and Indels**. A figure showing a heat map of sequence changes in SNPs and indels. Click here for file

Additional file 10 **Most and least variable genes**. A table showing results of Fisher's exact test for GO term enrichment in the most and least variable genes. Click here for file
