# Supplementary material for: Gene discovery in the horned beetle Onthophagus taurus
Source: BMC Genomics. 2010 Dec 14;11:703. doi: 10.1186/1471-2164-11-703 (PMC3019233; doi:10.1186/1471-2164-11-703)
Supplement: Additional file 2 — Onthophagus taurus repeated sequences. A table containing a summary of repeated sequences by RepeatMasker. [file 1471-2164-11-703-S2.DOC]

Additional file 2: *Onthophagus taurus* repeated sequences

|  | Number of elements | Length occupied | Percentage of sequences |
| --- | --- | --- | --- |
| Total bases masked: |  | 278062 | 1.05 |
| Retroelements | 76 | 14223 | 0.05 |
| SINEs: | 0 | 0 | 0.00 |
| LINEs: | 37 | 3996 | 0.02 |
| Penelope | 7 | 284 | 0.00 |
| CRE/SLACS | 0 | 0 | 0.00 |
| L2/CR1/Rex | 11 | 1403 | 0.01 |
| R1/LOA/Jockey | 2 | 126 | 0.00 |
| R2/R4/NeSL | 0 | 0 | 0.00 |
| RTE/Bov-B | 0 | 0 | 0.00 |
| L1/CIN4 | 0 | 0 | 0.00 |
| LTR elements: | 39 | 10227 | 0.04 |
| BEL/Pao | 0 | 0 | 0.00 |
| Ty1/Copia | 12 | 2282 | 0.01 |
| Gypsy/DIRS1 | 27 | 7945 | 0.03 |
| Retroviral | 0 | 0 | 0.00 |
| DNA transposons | 41 | 9612 | 0.04 |
| hobo-Activator | 2 | 200 | 0.00 |
| Tc1-IS630-Pogo | 0 | 0 | 0.00 |
| En-Spm | 0 | 0 | 0.00 |
| MuDR-IS905 | 0 | 0 | 0.00 |
| PiggyBac | 0 | 0 | 0.00 |
| Tourist/Harbinger | 0 | 0 | 0.00 |
| Other (Mirage,   P-elements, Transib) | 12 | 2633 | 0.01 |
| Rolling-circles | 0 | 0 | 0.00 |
| Unclassified: | 0 | 0 | 0.00 |
| Total interspersed repeats: |  | 23835 | 0.09 |
| Small RNA: | 67 | 15061 | 0.06 |
| Satellites: | 0 | 0 | 0.00 |
| Simple repeats: | 579 | 33036 | 0.12 |
| Low complexity: | 5435 | 206130 | 0.78 |
