# Supplementary figures and images for: Gene discovery in the horned beetle Onthophagus taurus
Source: BMC Genomics. 2010 Dec 14;11:703. doi: 10.1186/1471-2164-11-703 (PMC3019233; doi:10.1186/1471-2164-11-703)

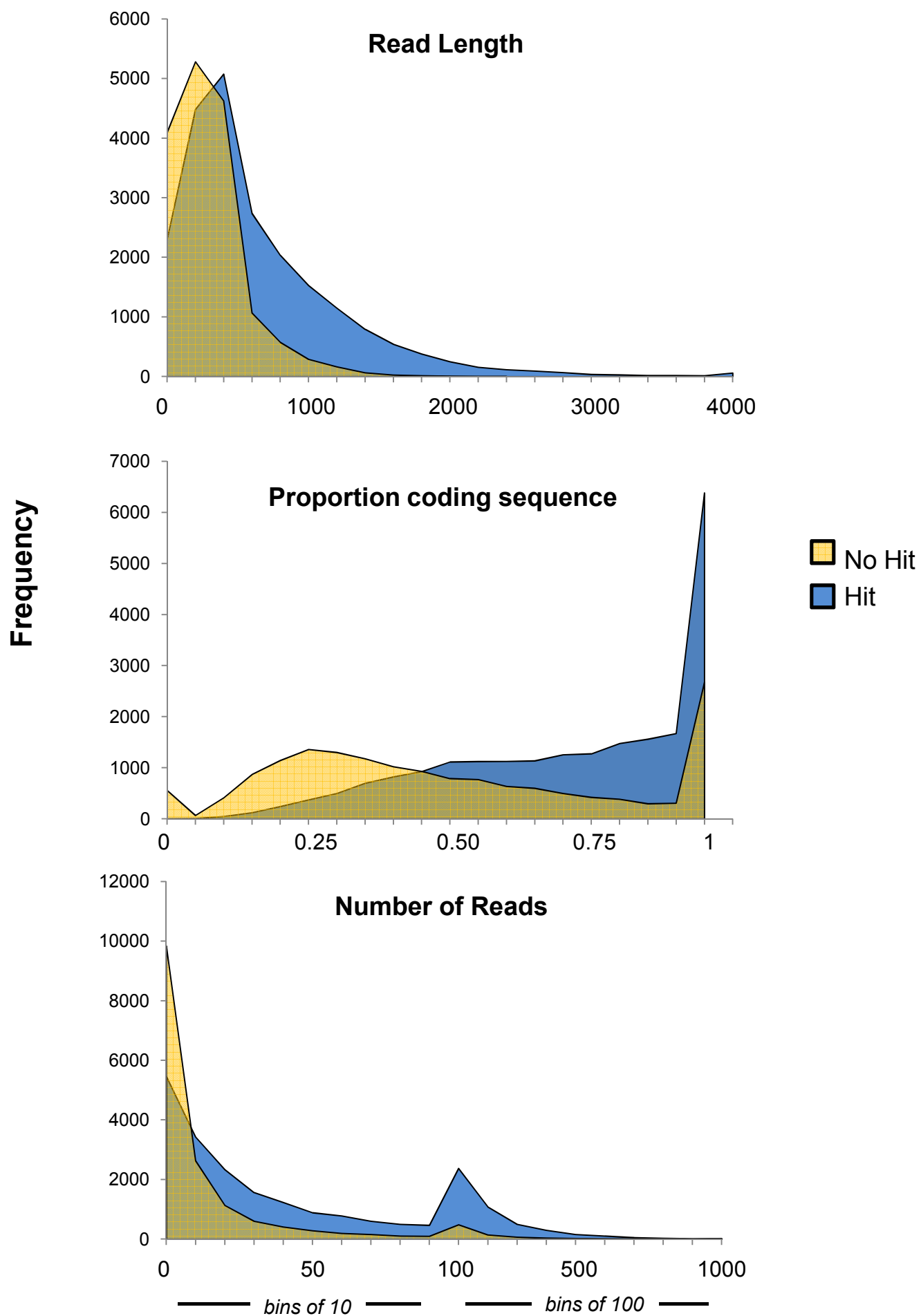

Supplement: Additional file 3 — Comparison of read quality between sequences with and without database hits. "Hit" refers to contigs with significant (e value < 10-5) match against the Tribolium genome and protein databases and/or the NCBI NR database (N = 21847 total). "No Hit" refers to sequences with no significant database match (N = 16203). Shown are histograms for contig length, the proportion of a contig that represents coding sequence, and total read number for a contig. [file 1471-2164-11-703-S3.PDF]
