# Supplementary material for: Gene discovery in the horned beetle Onthophagus taurus
Source: BMC Genomics. 2010 Dec 14;11:703. doi: 10.1186/1471-2164-11-703 (PMC3019233; doi:10.1186/1471-2164-11-703)
Supplement: Additional file 8 — Confidence distribution of sequence variants. A figure showing frequency histograms of the confidence scores of sequence variants. "Major allele" refers to the more common sequence variant, while "minor allele" refers to the rarer sequence variant. [file 1471-2164-11-703-S8.DOC]

Additional file 8: frequency histograms of the confidence scores of sequence variants.


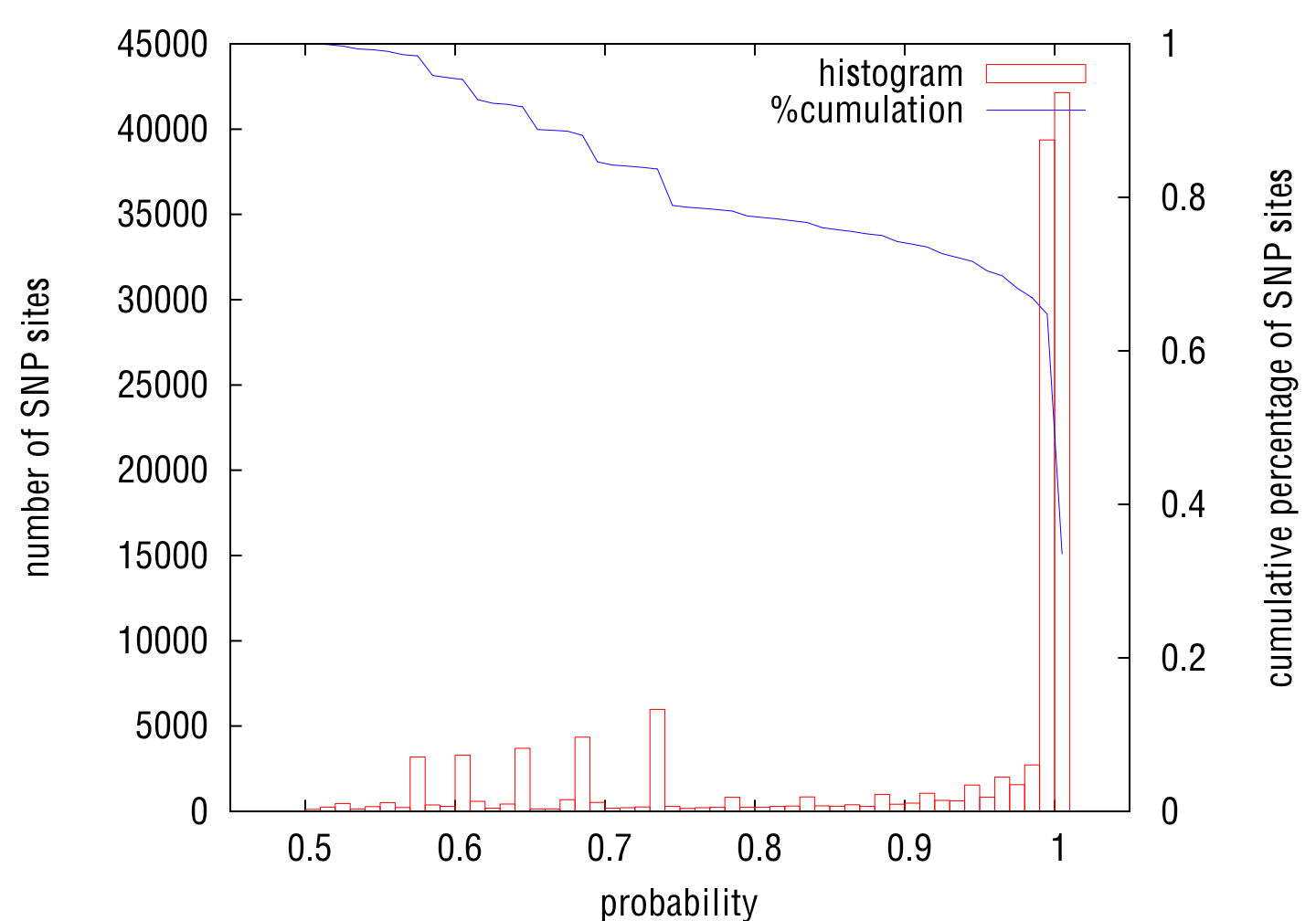


(a) SNPs


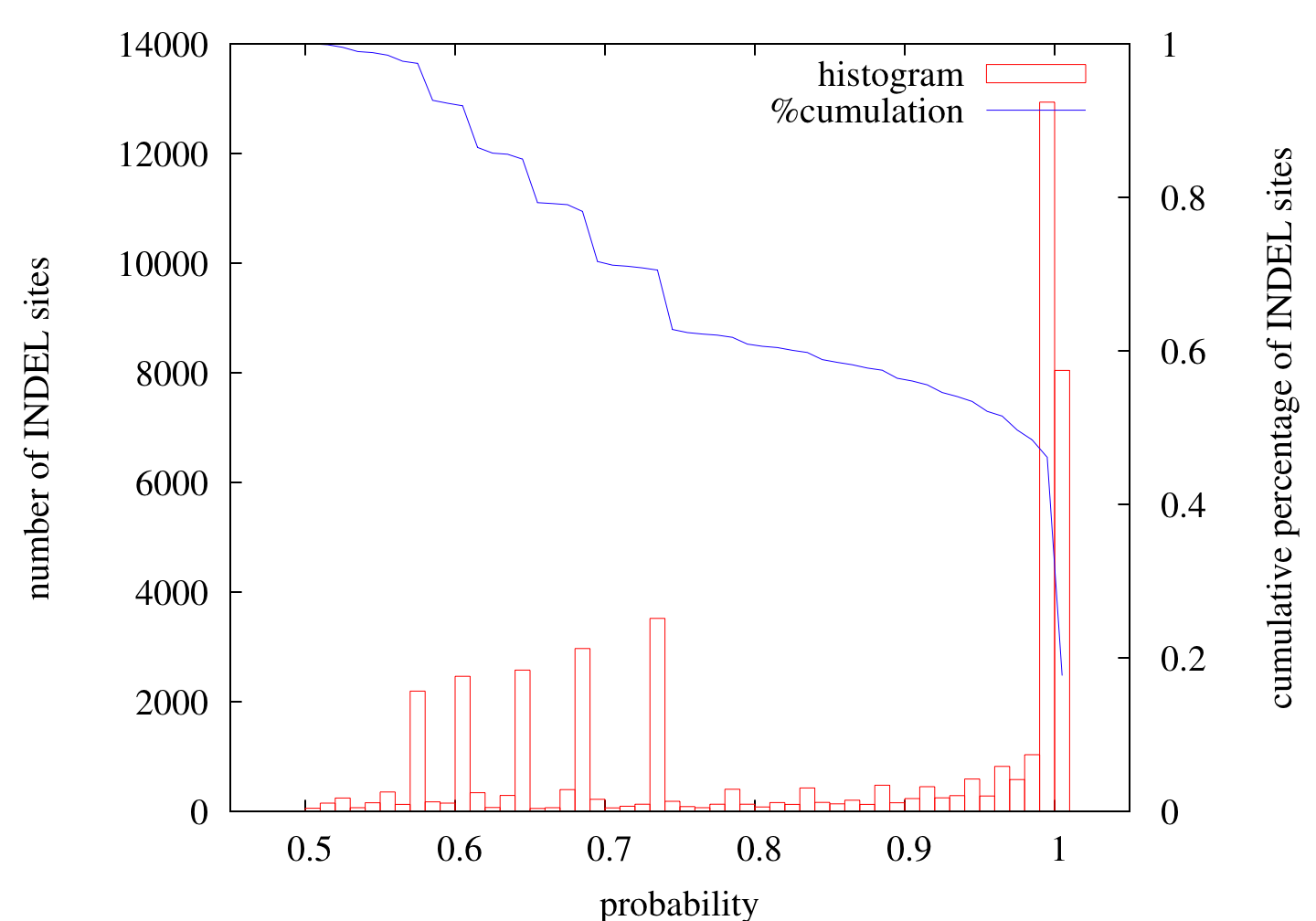


(b) INDELs

Supplementary Figure 1. Histogram of Probabilites of (a) SNPs and (b) INDELs.
