# Supplementary material for: Gene discovery in the horned beetle Onthophagus taurus
Source: BMC Genomics. 2010 Dec 14;11:703. doi: 10.1186/1471-2164-11-703 (PMC3019233; doi:10.1186/1471-2164-11-703)

Additional file 9: Sequence changes of SNPs and Indels where the X axis represents major allele bases and the Y axis represents minor allele bases.


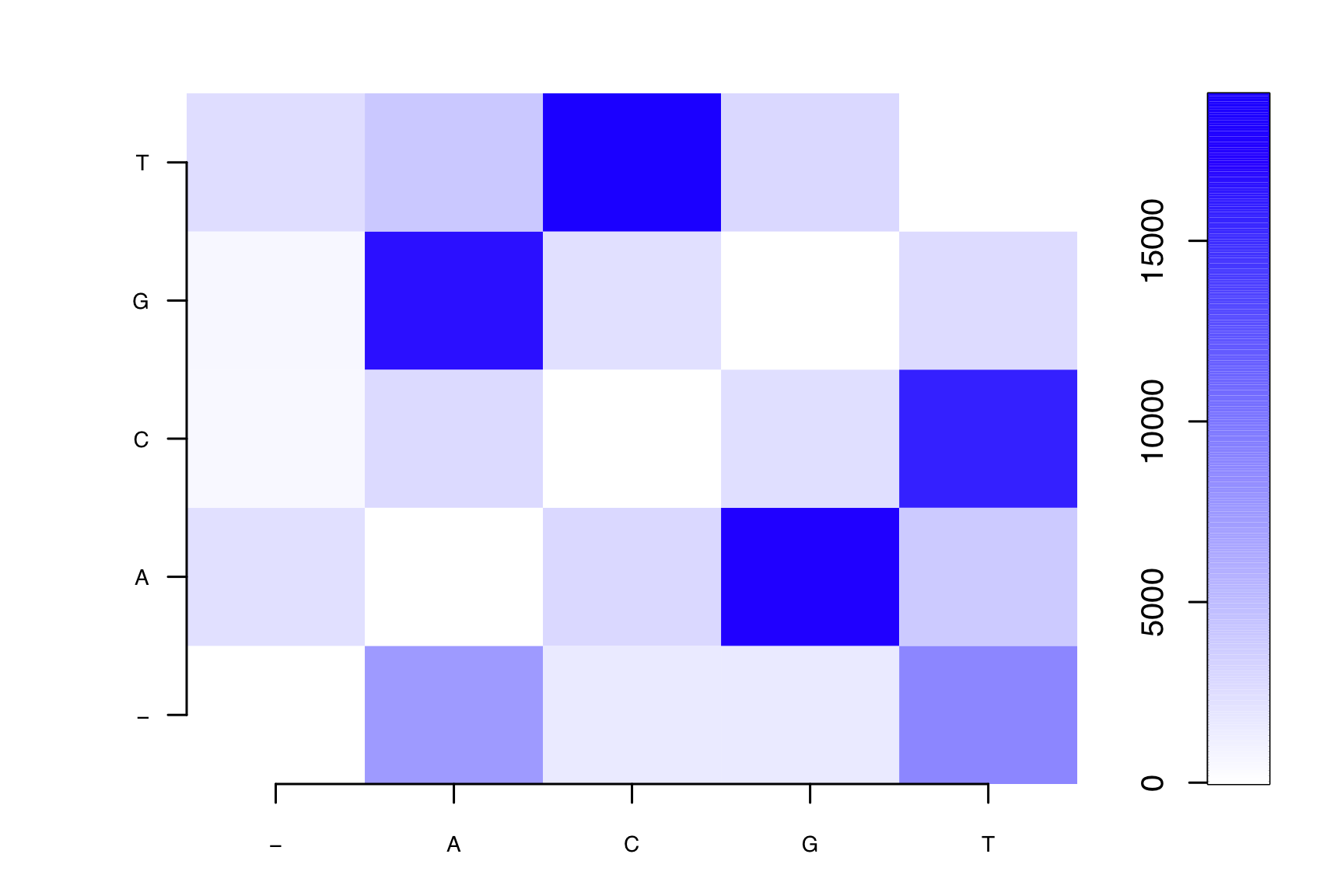

Supplement: Additional file 9 — Sequence changes of SNPs and Indels. A figure showing a heat map of sequence changes in SNPs and indels. [file 1471-2164-11-703-S9.DOC]
