# Supplementary material for: Gene discovery in the horned beetle Onthophagus taurus
Source: BMC Genomics. 2010 Dec 14;11:703. doi: 10.1186/1471-2164-11-703 (PMC3019233; doi:10.1186/1471-2164-11-703)
Supplement: Additional file 10 — Most and least variable genes. A table showing results of Fisher's exact test for GO term enrichment in the most and least variable genes. [file 1471-2164-11-703-S10.DOC]

**Additional file 10a.** ***Results of Fisher’s Exact Test for GO Term enrichment in the most variable genes*** (top 5% of genes ranked for residual SNP frequency).

| **GO Term** | **Name** | **FDR** |
| --- | --- | --- |
| GO:0003779 | actin binding | 0.00444714 |
| GO:0008092 | cytoskeletal protein binding | 0.00531464 |
| GO:0006519 | cellular amino acid and derivative metabolic process | 0.0316552 |

**Additional file 10b.** ***Results of Fisher’s Exact Test for GO Term enrichment in the least variable genes*** (lowest 5% of genes ranked for residual SNP frequency).

| **GO Term** | **Name** | **FDR** |
| --- | --- | --- |
| GO:0005811 | lipid particle | 1.69E-04 |
| GO:0019538 | protein metabolic process | 1.69E-04 |
| GO:0043229 | intracellular organelle | 3.26E-04 |
| GO:0048856 | anatomical structure development | 3.26E-04 |
| GO:0009653 | anatomical structure morphogenesis | 3.26E-04 |
| GO:0044424 | intracellular part | 5.08E-04 |
| GO:0005622 | intracellular | 5.08E-04 |
| GO:0043170 | macromolecule metabolic process | 7.43E-04 |
| GO:0043283 | biopolymer metabolic process | 7.43E-04 |
| GO:0032502 | developmental process | 8.00E-04 |
| GO:0007010 | cytoskeleton organization | 8.00E-04 |
| GO:0005198 | structural molecule activity | 0.00105201 |
| GO:0043226 | organelle | 0.00150379 |
| GO:0032991 | macromolecular complex | 0.00150379 |
| GO:0044267 | cellular protein metabolic process | 0.00150379 |
| GO:0044464 | cell part | 0.00150379 |
| GO:0007275 | multicellular organismal development | 0.00150379 |
| GO:0032501 | multicellular organismal process | 0.00150379 |
| GO:0043232 | intracellular non-membrane-bounded organelle | 0.00150379 |
| GO:0043228 | non-membrane-bounded organelle | 0.00150379 |
| GO:0005737 | cytoplasm | 0.00169015 |
| GO:0016043 | cellular component organization | 0.00169015 |
| GO:0030246 | carbohydrate binding | 0.00273839 |
| GO:0005856 | cytoskeleton | 0.00379816 |
| GO:0005488 | binding | 0.004444 |
| GO:0008219 | cell death | 0.00469066 |
| GO:0000166 | nucleotide binding | 0.00469066 |
| GO:0043412 | biopolymer modification | 0.00469066 |
| GO:0006464 | protein modification process | 0.00469066 |
| GO:0044237 | cellular metabolic process | 0.00469066 |
| GO:0016265 | death | 0.00469066 |
| GO:0044238 | primary metabolic process | 0.00642412 |
| GO:0043234 | protein complex | 0.00713636 |
| GO:0016301 | kinase activity | 0.00893138 |
| GO:0016772 | transferase activity, transferring phosphorus-containing groups | 0.00893138 |
| GO:0030528 | transcription regulator activity | 0.00920292 |
| GO:0008152 | metabolic process | 0.00920292 |
| GO:0006996 | organelle organization | 0.00941185 |
| GO:0009987 | cellular process | 0.00941185 |
| GO:0003700 | transcription factor activity | 0.00974016 |
| GO:0007049 | cell cycle | 0.0106758 |
| GO:0044260 | cellular macromolecule metabolic process | 0.0116746 |
| GO:0034960 | cellular biopolymer metabolic process | 0.0116746 |
| GO:0022900 | electron transport chain | 0.0135791 |
| GO:0022904 | respiratory electron transport chain | 0.0135791 |
| GO:0055114 | oxidation reduction | 0.0135791 |
| GO:0016773 | phosphotransferase activity, alcohol group as acceptor | 0.0146323 |
| GO:0004672 | protein kinase activity | 0.0146323 |
| GO:0005576 | extracellular region | 0.0151766 |
| GO:0044444 | cytoplasmic part | 0.0211504 |
| GO:0005829 | cytosol | 0.0212599 |
| GO:0009607 | response to biotic stimulus | 0.0212599 |
| GO:0043231 | intracellular membrane-bounded organelle | 0.0212599 |
| GO:0043227 | membrane-bounded organelle | 0.0212599 |
| GO:0008092 | cytoskeletal protein binding | 0.0212599 |
| GO:0048869 | cellular developmental process | 0.0363741 |
| GO:0030154 | cell differentiation | 0.0363741 |
| GO:0003723 | RNA binding | 0.0422741 |
| GO:0030529 | ribonucleoprotein complex | 0.0455938 |
| GO:0005840 | ribosome | 0.0455938 |
| GO:0000003 | reproduction | 0.0455938 |
